# New Method of Constructing Rubber Plates

**Published:** 1869-02

**Authors:** 


### ARTICLE VI.

### *New Method of Constructing Rubber Plates.*

A few weeks since, a man by the name of Myres, claiming to be the agent of Messrs. Stuck & Owen, of Ryan, Ohio, called upon some of the members of the profession here, in St. Louis, the object of his visit being to give instruction in, and to sell the right to use a new method of manufacturing rubber plates.

This important discovery, as he claimed, of Messrs. S. & O., had just been patented by them, and they would now give the profession notice of their improvement through the medium of a dental journal. Being anxious to give the profession in St. Louis the benefit of the discovery as soon as possible, Messrs. S. & O. had sent him to act as instructor and agent.

The advantages of this new method, as set forth in his circular, were very great, while the fee for instruction and right was very small—only $20

To the Dental Colleges he would give instruction and right gratis, those receiving the favor binding themselves in writing not to divulge the secret.

The following, taken from the circular, will give the readers of the Journal some faint idea of Messrs. S. & O.'s discovery, and its advantages :

"A new method of manufacturing rubber plates, by which they are taken from the flask, polished on both sides, and of uniform thickness. Some of the superior advantages of this method over the old process are a more perfect fit or adaptation, producing a large increase in mastication: and being of uniform thickness, like gold plate, it does away with the unnatural incumbrance in the mouth, while they are readily preferred by lovers of cleanliness, and universally acknowledged by all classes to be a nearer approximation to what an artificial plate should be. It saves all liabilities of scraping or polishing a hole in plates, and last, but not least, it saves one-half of the labor of finishing. It is the only scientific method known, to prevent a manifest tendency of the plate to rest on the arch or palate of the mouth, before striking the alveolar, which causes them to rock with the least pressure and drop from the mouth. Attention is also invited to our method for re-setting or attaching teeth to plates when broken off, by which, after they are ground to fit, the fastening can be made in ten minutes, so that it cannot be noticed that they were broken.

Comment on this process is unnecessary. * * * This leaves the plate as strong and nice in appearance as before mending, and is as remunerative as any portion of dentistry. Five dollars for printed instructions in this alone has been received from different parts of the United States, England, and France. Our method also, for making metalic casts or dies, from plaster impressions, taken from the mouth, by which, instead of pouring in the plaster, metal is poured in the impression while in cups, and a metalic model, which is free from the liabilities and objections of the plaster model is obtained. * * * To the operator, who makes only an occasional metal plate, the price asked for the whole of these instructions is of but little consideration, compared with the value of this process alone. Prominent operators in Cincinnati, after receiving instructions, (not before), said that $25 would be reasonable for this alone," etc.

Appended to this circular were the names of several wellknown operators, who were represented as having recommended it to the amount of the fee for instructions.

With these superior advantages, as set forth in the circular, and by Mr. Agent, backed by Drs. Berry, Merideth, Taylor and others of Cincinnati, a class of instruction was soon formed, instructions imparted, contracts signed, receipts of deeds given; and with the promise to send to each member of the class, immediately upon his return to Ohio, a deed of right to use, some chemically pure sheet tin, and a hardened copper wheel—articles used in the new process—Mr. Agent shook the dust from his feet and departed. The material was not at hand or procurable, at the time the instruction was being given, hence no opportunity was given to test the merits of the great improvements.

In due time, some tin was received, as promised, but no deeds, no copper wheels, hence but part of the instruction could be made available. In justice to those who have taken instruction, Messrs. S. & O. should make their agent fulfil his contract.

We were not one of the *instructed*, and consequently, are not bound to keep the secret. We therefore propose to give the reader the benefit of what we know of this great discovery, not to harm Messrs. S. & O., they being protected, so Mr. Agent tells, us by letters patent, but to give to the profession all there is new in this department.

The method of manufacturing rubber plates is as follows: The impression is taken with plaster alone, using an ordinary metalic impression cup, altered for the purpose by cutting a hole $\frac{5}{8}$ to $\frac{3}{4}$ of an inch square, out of the centre. With the cup thus prepared, take your impression, and on removing it from the mouth, varnish and oil lightly Prepare a little plaster, and pour it over the central portion of your impression, covering about $\frac{3}{4}$ of the palatine portion. When sufficiently hard, remove it, and, with a small knife, cut a hole through the centre of the impression, corresponding with the hole in the cup. Build up the edges of your impression with plaster, say, $\frac{1}{2}$ inch, letting the plaster extend across the arch, back of where you wish the plate to come, thus forming a cup for the receipt of the metal for your die. Return the piece of plaster which was cast over the central portion to its position on the impression, and retain it there while you fill up the hole through the cup with moulding sand. Remove the piece of plaster from the central portion. The impression is now ready to receive the metal.

The metal used is block tin. Melt at low heat, and pour as soon as sufficiently fluid. The gas and steam generated in pouring the hot tin upon the damp plaster of the impression, is supposed to find its exit through the sand in the centre. Upon this metalic model make your trial plate of sheet lead. The sheet lead used to line tea chests is recommended for this purpose. Use two, three, or more thicknesses, dependent upon the thickness desired for the plate. Take the articulation as for any other style. The teeth should not be ground to fit the model too closely, as they are liable to fracture in closing the flask.

Having the work ready to flask, cover the surface of the model with a sheet of chemically pure tin, which should be well burnished down, to prevent the admission of anything beneath it. All creases or folds on the surface should be well burnished out, and then polished by rubbing briskly with a small tuft of cotton. The object of this covering of tin foil is two-fold: it compensates for the shrinkage of the metalic model, and should, therefore, vary in thickness, dependent upon size and characteristics of the mouth. It gives the polished surface to the plate; hence, the surface of the tin should be smooth and well polished.. A ridge may be raised round the border of the plate, if desired, corresponding with the thickness of the sheet tin used, by not allowing the tin to extend up to the edge of the plate. Place the case upon the model, and wax up the rim. Remove all surplus wax from the lingual surface, leaving none over the palatine portion, and only what is necessary at any other point; cover the lingual surface with a sheet of pure tin, letting it extend far beyond the border of the teeth. The case is now ready for the flask. Pack and vulcanize same as other styles. Take from the flask, polish the rim and edges of the plate, and the case is complete.

To repair a case, when a block has been broken off, grind the new block to fit the surface of the rubber, as left by the old block, as nearly as possible; drill or cut in the surface of the rubber dove-tailed holes or slots; with a hardened copper wheel, cut grooves in several directions in the lingual surface of the block. Take a little fusible metal, melting it at a very low temperature, fill the holes and slots in the rubber, and the grooves in the block, using a little more than is necessary to fill them; taking the block in one hand, the case in the other, warm them over a spirit lamp sufficiently to fuse the metal, then place the block in position, press it gently into place, forcing out the surplus metal; hold it till the fastening cools, and it is completed.

We think it will be found a very difficult matter to get up a sharp metalic die, without drying the impression a little before casting the die. The use of sheet lead for trial plates, as directed by Messrs. S. & O., was recommended for that purpose, in the first instructions given in rubber work. We hope some one will take the hints suggested by this method of Messrs. S. & O., and get up something more practical.—*Missouri Dental Journal.*

---

